# Artificial neural networks improve and simplify intensive care mortality prognostication: a national cohort study of 217,289 first-time intensive care unit admissions

**DOI:** 10.1186/s40560-019-0393-1

**Published:** 2019-08-16

**Authors:** Gustav Holmgren, Peder Andersson, Andreas Jakobsson, Attila Frigyesi

**Affiliations:** 1 Centre for Mathematical Sciences, Mathematical Statistics, Lund University, Sölvegatan 18, Lund, SE-22362 Sweden; 2 Department of Clinical Medicine, Anaesthesiology and Intensive Care, Lund University, Lund, SE-22185 Sweden; 3 Skåne University Hospital, Intensive and Perioperative Care, Lund, SE-22185 Sweden

**Keywords:** Machine learning, Artificial intelligence, Artificial neural networks, Intensive care, Critical care, Mortality, Prediction, Survival

## Abstract

**Purpose:**

We investigated if early intensive care unit (ICU) scoring with the Simplified Acute Physiology Score (SAPS 3) could be improved using artificial neural networks (ANNs).

**Methods:**

All first-time adult intensive care admissions in Sweden during 2009–2017 were included. A test set was set aside for validation. We trained ANNs with two hidden layers with random hyper-parameters and retained the best ANN, determined using cross-validation. The ANNs were constructed using the same parameters as in the SAPS 3 model. The performance was assessed with the area under the receiver operating characteristic curve (AUC) and Brier score.

**Results:**

A total of 217,289 admissions were included. The developed ANN (AUC 0.89 and Brier score 0.096) was found to be superior (*p* <10^−15^ for AUC and *p* <10^−5^ for Brier score) in early prediction of 30-day mortality for intensive care patients when compared with SAPS 3 (AUC 0.85 and Brier score 0.109). In addition, a simple, eight-parameter ANN model was found to perform just as well as SAPS 3, but with better calibration (AUC 0.85 and and Brier score 0.106, *p* <10^−5^). Furthermore, the ANN model was superior in correcting mortality for age.

**Conclusion:**

ANNs can outperform the SAPS 3 model for early prediction of 30-day mortality for intensive care patients.

## Introduction

Outcome prediction on admission to the intensive care unit (ICU) is a difficult task as patients are admitted with a wide array of diseases with varying severity in addition to patients’ diversity in terms of age and comorbidities. In this study, we investigate if the current gold standard of early (within 1 h of admission) ICU-scoring, the Simplified Acute Physiology Score (SAPS 3) [1, 2] could be improved using artificial neural networks (ANN).

**Fig. 1:**
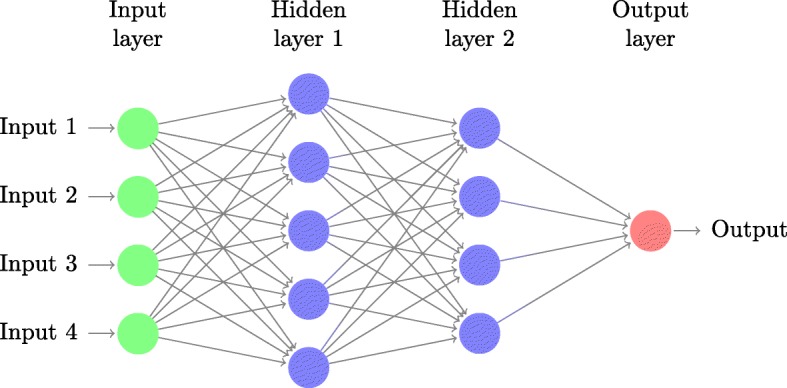
ANN. A schematic artificial neural network (ANN) with two hidden layers and a single neuron output

An ANN is a collection of nodes or artificial neurons, which loosely model the neurons of the brain. Each connection or edge, like the synapses in a biological brain, can transmit a signal from one node to another (see Fig. 1). A node that receives a signal processes it and subsequently conducts it outwards to other conjoined nodes. The signal between nodes is typically a real number, and the output of each artificial neuron is computed by some non-linear function of the sum of its inputs. Artificial neurons and edges typically have weights that adjust as learning proceeds. The weight increases or decreases the strength of the signal at a connection [3].

Advances in computing speed and the development of efficient algorithms have led to a renaissance for machine learning techniques such as ANNs during the last decade. The use of machine learning has proven to be valuable in a wide variety of medical fields, from the interpretation of cardiac magnetic resonance imaging for mortality prediction of pulmonary hypertension to detecting skin cancer [4, 5]. Machine learning has also been found to be a promising technique in prognostication of the critically ill but only in conjunction with data available after 24 h and comparing with the Acute Physiology And Chronic Health Evaluation (APACHE) model. In a study from 2015, Pirracchio et al. found that an ensemble of machine learning techniques could improve ICU prediction [6]. Similarly, in Kim et al. [7], the authors used different machine learning algorithms to estimate ICU mortality from data collected within the first 24 h of ICU admission.

Current ICU prediction models such as the APACHE, used for scoring within the first 24 h, the Mortality Prediction Model (MPM), used for scoring on admission or after 24 hours, and the SAPS 3 [8] are based on multivariable logistic regression models. The SAPS 3 uses characteristics such as comorbidities before ICU admission, the reason for ICU admission, physiological parameters, and laboratory findings within 1 h of ICU admission to calculate an estimated mortality risk (EMR) [1, 2]. The SAPS 3 has been re-calibrated several times to improve its performance [9]. To our knowledge, machine learning has not yet been used to improve early prognostication (prospectively registered within the first hour of admission) or using the massive data repositories of a national intensive care registry.

The aim of this study was to improve the 30-day mortality prognostication within the first hour of ICU admission using ANN modelling on data prospectively gathered within the first hour of admission (for SAPS 3 prognostication), as well as to identify the smallest possible subset of the more-than-twenty SAPS 3 parameters that can retain the same performance as the SAPS 3 model.

## Materials and methods

We identified all first-time adult ICU admissions (excluding cardiothoracic ICU admissions as these use a different scoring system) with follow-ups for at least 30 days during 2009–2017 from the Swedish Intensive Care Registry (SIR). Both SAPS 3 parameters and 30-day mortality were used in this study. Physiological parameters and laboratory findings were prospectively recorded within 1 h of ICU admission, and an estimated mortality ratio (EMR) was calculated according to the latest Swedish calibration from 2016. This calculation estimates the 30-day mortality, in contrast to the original SAPS 3 model, which estimates the in-hospital mortality [9]. In Sweden, the Reaction Level Scale (RLS85) is often used instead of the more widespread Glasgow Coma Scale (GCS). For the studied admissions, 80% had RLS85 recorded, 20% had GCS recorded, whereas 2.5% had neither. Instead of translating GCS to RLS85, we chose to transform both scales to the central nervous system (CNS) scale used by APACHE II [10] and then use CNS scores in our ANN. See Table 1 for a comprehensive list of the SAPS 3 parameters.

**Table 1:** Descriptive statistics

	Training set	Test set	*p* value	Survivors	Non-survivors	*p* value
Number of patients	181,075	36,214		177,185	40,104	<0.001
Women (%)	43.5	42.9	0.032	43.6	42.6	<0.001
Mean LOS (days)	2.49 (0.52–2.32)	2.50 (0.52–2.34)	0.29	0.383 (0.208–0.841)	0.516 (0.210–1.315)	<10^−15^
ICU mortality (%)	8.8	8.8	0.85	0.00109	0.47	<10^−15^
30-day mortality (%)	18.5	18.5	0.87	0	100	<10^−15^
Median SAPS 3 score	53 (42–65)	52 (41–64)	0.30	49 (39–59)	70 (61–80)	<10^−15^
Median EMR_SAPS 3_	0.100 (0.027–0.280)	0.090 (0.024–0.261)	0.30	0.065 (0.018–0.176)	0.382 (0.208–0.589)	<10^−15^
Box I						
Median age (years)	65 (48–76)	65 (48–76)	0.66	63 (43–73)	74 (66–82)	<10^−15^
Comorbidities						
Cancer therapy (%)	4.7	4.8	0.51	4.1	7.4	<10^−15^
Chronic HF (%)	5.5	5.5	1	4.0	11.8	<10^−15^
Haematological cancer (%)	1.7	1.7	0.75	1.2	4.0	<10^−15^
Cirrhosis (%)	1.8	1.8	0.64	1.5	3.5	<10^−15^
AIDS (%)	0.092	0.102	0.62	0.092	0.100	0.71
Cancer (%)	8.4	8.4	0.88	7.4	12.8	<10^−15^
Mean LOS before ICU (days)	1.8 (0–1)	1.7 (0–1)	0.12	1.6	2.8	<10^−15^
Location before ICU						
Operation (%)	11.4	11.3	0.50	12.5	6.8	<10^−15^
Emergency room (%)	53.1	53.2	0.65	54.8	45.8	<10^−15^
Other ICU (%)	2.6	2.7	0.57	2.4	3.4	<10^−15^
Other (%)	30.0	29.8	0.57	27.4	41.1	<10^−15^
Vasoactive drugs before ICU (%)	12.8	12.8	0.73	11.3	19.4	<10^−15^
Box II						
Unplanned ICU admission (%)	92.7	92.6	0.60	92.0	96.0	<10^−15^
Reason for ICU admission						
Basic and observational (%)	14.0	14.3	0.10	16.4	3.8	<10^−15^
Neurological (%)	46.3	46.2	0.66	46.6	44.7	<10^−11^
Cardiovascular (%)	45.3	45.8	0.068	42.5	57.8	<10^−15^
Respiratory (%)	46.7	46.9	0.46	45.3	53.2	<10^−15^
Hepatic (%)	18.1	18.3	0.46	19.7	11.0	<10^−15^
Digestive (%)	27.5	27.8	0.29	29.1	20.8	<10^−15^
Renal (%)	27.6	27.9	0.23	27.6	27.6	0.97
Metabolic (%)	33.0	33.2	0.48	34.0	28.8	<10^−15^
Haematological (%)	18.9	19.4	0.014	20.5	12.1	<10^−11^
Trauma (%)	9.8	9.7	0.40	10.6	6.4	<10^−15^
Other (%)	10.5	10.6	0.50	11.1	8.0	<10^−15^
Surgical status at ICU admission						
No surgery (%)	79.7	79.8	0.60	78.4	85.4	<10^−15^
Scheduled surgery (%)	9.0	9.1	0.88	10.2	3.7	<10^−15^
Emergency surgery (%)	11.2	11.1	0.41	11.3	10.8	0.0048
Anatomical site of surgery						
Transplantation surgery (%)	0.40	0.44	0.25	0.49	0.047	<10^−15^
Isolated trauma (%)	0.60	0.59	0.89	0.60	0.61	0.90
Multiple trauma (%)	0.37	0.40	0.39	0.43	0.15	<10^−15^
Cardiac surgery (%)	0.41	0.48	0.070	0.44	0.32	0.00085
Neurosurgery (%)	1.2	1.2	0.26	1.2	1.2	0.70
All other types of surgery (%)	17.9	17.8	0.65	19.0	12.8	<10^−15^
Acute infection at ICU admission						
Nosocomial (%)	2.7	2.8	0.50	2.3	4.5	<10^−15^
Respiratory (%)	10.6	11.0	0.059	8.9	18.7	<10^−15^
Box III						
Median GCS	15 (11–15)	15 (11–15)	0.082	15 (13–15)	10 (3–14)	<10^−15^
Median total bilirubin (*μ*mol/L)	10 (6–17)	10 (6–17)	0.70	10 (6–16)	11 (7–20)	<10^−15^
Mean max. temperature (^∘^C)	36.8 (36.2–37.5)	36.8 (36.2–37.5)	0.94	36.9 (36.3–37.5)	36.5 (35.8–37.4)	<10^−15^
Median max. creatinine (*μ*mol/L)	84 (64–123)	84 (64–123)	0.88	80 (63–112)	110 (76–175)	<10^−15^
Mean max. heart rate (bpm)	98 (80–114)	98 (80–114)	0.52	97	102	<10^−15^
Median max. leukocyte count (×10^9^/L)	11.1 (8.0–15.6)	11.2 (8.0–15.6)	0.80	10.9 (7.9–15.0)	12.6 (8.6–17.7)	<10^−15^
Median min. pH	7.36 (7.29–7.42)	7.36 (7.29–7.42)	0.22	7.37 (7.30–7.42)	7.31 (7.20–7.40)	<10^−15^
Median min. platelet count (×10^9^/L)	222 (165–287)	222 (164–287)	0.89	225 (169–287)	208 (142–286)	<10^−15^
Median min. systolic BP (mmHg)	110 (90–130)	110 (89–130)	0.12	111 (90–133)	92 (70–120)	<10^−15^
Oxygenation						
Over pressure ventilation (%)	30.5	30.0	0.093	25.6	51.6	<10^−15^
Median FiO_2_	0.40 (0.30–0.60)	0.40 (0.30–0.60)	0.18	0.40 (0.30–0.50)	0.50 (40–80)	<10^−15^
Median PaO_2_ (kPa)	11.9 (9.4–15.9)	11.8 (9.4–15.8)	0.43	12.0 (9.7–16.0)	11.0 (8.7–15.3)	<10^−15^

Mean values, medians, and modes (always with interquartile ranges) and *p* values from Wilcoxon Rank test and *χ*^2^ test, as applicable *LOS* length of stay

In order to select an appropriate network, we constructed 200 single-output ANNs using two hidden layers, where the number of nodes in each layer was log-sampled between 5 and 400. These networks were constructed using TensorFlow [11], which is a Python-based open-source machine learning framework developed by Google LLC (Mountain View, USA). To improve convergence, training speed, and accuracy, we normalise each layer using batch normalisation, so that the output of these have zero mean and unit variance [12]. The loss function was optimised using the Adam implementation of stochastic gradient descent (SGD) [13], using a learning rate of 0.001. This choice was made as stochastic gradient descent-based methods are the current state-of-the-art technique for optimising ANN loss functions [14]. Regularisation was performed using log-sampled weight decay with the decay parameter, *λ*, ranging from 10^−7^ to 10^−3^. To increase feature selection capabilities and to further improve regularisation, dropout was used, where *p* was log-sampled from 5% to 20% on the input layer and 40% to 60% on the hidden layers [15]. The network was trained for 100 epochs with a batch size of 512 using ReLU activation functions on the hidden layers [14]. In order to find the selected network, fivefold cross-validation was used, which yielded the hyper-parameters of our network: 158 first-layer nodes and 67 second-layer nodes with a weight decay of *λ*=5.04×10^−6^. The dropout rates were 0.073 (input) and 0.501 (hidden). Data were randomly divided into six portions, with one portion set aside for independent validation purposes (the test set). Simple mean and mode substitution turned out to perform just as well as the more advanced methods for imputation, such as autoencoders [16].

To evaluate the performance of the ANN model, we examined the receiver operating characteristic (ROC) curve, which plots sensitivity, against 1-specificity, for various threshold settings. We used the area under the ROC curve (AUC) as a performance measure [17]. Differences in AUC were tested for with the method of DeLong et al. [18]. Furthermore, we computed the Brier score, which is a measure of the *calibration* of a set of probabilistic predictions; in effect, it is the mean squared error of the forecast [19]. Differences in Brier scores were tested with an approximate permutation test with 50,000 permutations [20]. We evaluated our ANN models with the AUC of the ROC and the Brier score for the calibration error on the test set. The ratio between the 30-day mortality and the EMR is the standardised mortality ratio (SMR), which is a morbidity-adjusted mortality measure. The SMR is only interesting as a group measure, as individual SMRs are either 0 (if the individual has not survived) or $\text {EMR}_{i}^{-1}$, where EMR_*i*_ is the EMR of individual *i* (who has survived). However, a way of defining an individual (or local) SMR is using smoothing techniques. We applied local polynomial regression using the default settings of the loess function of R [21] on mortality and EMR (and then interpolated evenly over the whole range). We subsequently calculated the ratio of the smoothed mortality and the smoothed EMR to obtain smoothed (local) estimates of SMR [22]. One possible interpretation of the SMR is that the closer the SMR is to 1, the better the EMR prognosticates the mortality.

## Results

**Fig. 2:**
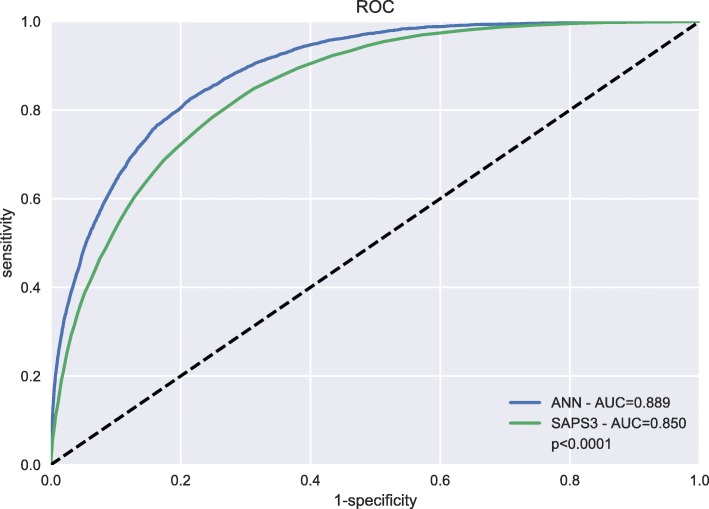
ROC. Receiver operating characteristic (ROC) curve for the artificial neural network (ANN) model and Simplified Acute Physiology Score (SAPS 3) model showed improved area under curve (AUC)

**Fig. 3:**
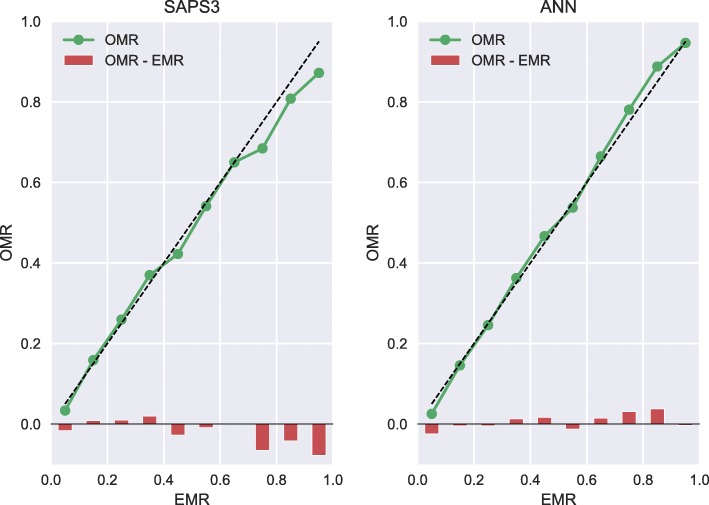
Calibration. Calibration curves (observed mortality ratio (OMR) versus expected mortality ratio (EMR)) for the Simplified Acute Physiology Score (SAPS 3) model and the artificial neural network (ANN) model demonstrated improved calibration (Brier score 0.096 vs. 0.110, *p* <10^−5^) in the high EMR range (0.7–1) for the ANN model

**Fig. 4:**
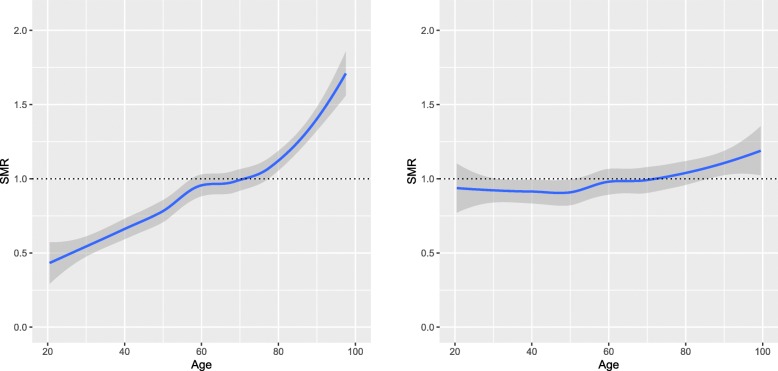
Age. Standardised mortality ratio (SMR) as a function of age for the Simplified Acute Physiology Score (SAPS 3) model (left panel) and the artificial neural network (ANN) model (right panel) for the test set (*n* = 36,214). The ANN model was superior in correcting for age as a prognostic factor (the single most important prognostic factor) as compared to SAPS 3. SMR is shown with a 95% confidence interval

A total of 217,289 first-time admissions were identified, of which ^1^ /_6_th (*n* = 36,214) were randomly allocated to the test set whereas ^5^ /_6_th (*n* = 181,075) were randomly allocated to the training set. The median age was 65 years (interquartile range, IQR 48–76 years), while the median SAPS 3 score was 53 (IQR 42–65) and 30-day mortality was 18.5%. Baseline characteristics, including SAPS 3 parameters of the study population, are shown in Table 1. There were no differences in the SAPS 3 parameters between the test set and the training set (after correction for multiple testing) in any of the parameters shown in Table 1. All performance calculations were based on the separate test set of 36,214 patients. Our ANN model outperformed the SAPS 3 model in both AUC (0.89 vs. 0.85, *p* <10^−15^) and Brier score (0.096 vs. 0.110, *p* <10^−5^) in predicting 30-day mortality (see Figs. 2 and 3). In Fig. 3, we see that the calibration error (that is the difference between OMR and EMR) in the high EMR range (0.7 – 1) was reduced in the ANN model. The improvement in AUC using the ANN model over the SAPS 3 model for different primary ICU diagnoses can be seen in Table 2. The ANN model outperformed the SAPS 3 model for all the top primary diagnoses. In our study, an eight-parameter subset of the SAPS 3 parameters was the smallest subset that achieved better performance than the SAPS 3 model. The eight parameters were (in order of importance for AUC) age, level of consciousness, neurological cause, cardiovascular cause, cancer, temperature, pH, and leukocytes. The eight-parameter model had an AUC of 0.851 (95% CI 0.845–0.857) and a Brier score of 0.106 (95% CI 0.106–0.107). In Fig. 4, the SMR is displayed as a function of age, the most important prognostic factor. The ANN model was superior in correcting mortality (with respect to age as a prognostic factor) compared to the SAPS 3 model, which underestimated the mortality in the elderly ICU population. Conversely, the SAPS 3 model overestimated the mortality in the younger ICU population.

**Table 2:** The performance of the SAPS 3 model and the ANN model for different primary ICU diagnoses based on the test set (*n* = 36,214)

	Number of patients	AUC of SAPS 3	AUC of ANN	*p* value
Test set	36,214	0.850 (0.846–0.855)	0.889 (0.885–0.893)	<10^−15^
Cardiac arrest	1,651	0.858 (0.835–0.881)	0.893 (0.875–0.912)	<10^−7^
Septic shock	1,481	0.846 (0.821–0.870)	0.889 (0.869–0.909)	<10^−8^
Respiratory failure	1,467	0.830 (0.804–0.856)	0.878 (0.855–0.900)	<10^−8^
Gastrointestinal haemorrhage	1,324	0.878 (0.858–0.900)	0.910 (0.892–0.927)	<10^−5^
SIRS	1,320	0.836 (0.811–0.862)	0.884 (0.863–0.906)	<10^−8^
Trauma	1,301	0.844 (0.820–0.869)	0.882 (0.860–0.903)	<10^−5^
Bacterial pneumonia	1,173	0.856 (0.830–0.882)	0.895 (0.874–0.916)	<10^−7^
Seizures	797	0.847 (0.814–0.880)	0.892 (0.865–0.918)	<10^−4^
Head injury	760	0.833 (0.796–0.869)	0.888 (0.860–0.916)	<10^−5^

Mean, 95% confidence intervals, and *p* values were obtained using the method of DeLong [18] *SIRS* Systemic Inflammatory Response Syndrome

## Discussion

We have shown that a well-designed neural network model can outperform the SAPS 3 model in the prediction of 30-day mortality while using the same parameters obtained within 1 h of admission. The ANN model was better with regards to both sensitivity and specificity, as measured by the AUC of the ROC curve (0.89 vs. 0.85, *p* <10^−15^) and notably in the calibration (Brier score of 0.106 vs. 0.093; *p* <10^−5^). As seen in Fig. 3, the ANN model was better in predicting 30-day mortality in the sickest patients, to be specific those with a very high EMR over 0.70. We noted in Fig. 4 that the ANN model was superior in correcting the most important prognostic factor, namely age. This single improvement in detecting a nonlinear relationship may very well have been the major contributor to the improved performance of the ANN model. The improvement in AUC using the proposed ANN model over the SAPS 3 model varied for different diagnoses, as shown in Table 2. However, it is worth noting that the proposed ANN model outperformed the SAPS 3 model for all considered cases. As can be seen in the table, the poorer the performance in the SAPS 3 model, the bigger the improvement in the proposed ANN model. For example, in respiratory failure, the SAPS 3 model performs less well with an AUC of 0.83, which improved to 0.88 when using the ANN model. Conversely, in gastrointestinal haemorrhage, the SAPS 3 model performs well, with an AUC of 0.88, which is then only marginally improved to 0.91 when using the ANN model. In our study, an eight-parameter subset of the SAPS 3 parameters was the smallest subset that achieved better performance than the SAPS 3 model. This finding suggests the possibility of using a simple ANN model in the place of the SAPS 3 model, which would then require less resources and would increase the likelihood of successful registrations, something which would be optimal when introducing a new national ICU registry. An interesting comparison can be made with Granholm et al. [23], who developed a seven-parameter logistic regression model using parameters registered up to 24 h before and after admission for 90-day mortality prediction of general ICU admissions and severe sepsis/septic shock achieving an AUC of 0.72 (95% CI 0.71–0.74). Our eight-parameter ANN model used parameters registered within 1 h of admission achieved an AUC of 0.85, clearly indicating the superiority of machine learning for complex data. Pirracchio and colleagues used the publicly available MMIC-II database that consists of data on 24,508 ICU patients at the Beth Israel Deaconess Medical Center in Boston, USA [6]. They used a super learner algorithm that performs at least as well as the best performing algorithm of its 12 algorithms—one of which was an ANN. Their finding was that a random forest algorithm performed best, and they reached a cross-validated AUC of 0.88 (95% CI 0.87–0.89), as compared to 0.82 reached by APACHE II. In Pirracchio’s study, they had access to SAPS II data and APACHE II data, both of which are registered within the first 24 h of admission (in contrast with SAPS 3 that only use data from the first hour). It is significant to note that the AUC should be higher, as it is considerably easier to prognosticate mortality with data obtained within 24 h than it is within 1 h of ICU admission. Kim and colleagues compared a range of machine learning techniques for the identification of ICU mortality with APACHE III, using data recorded within the first 24 h, making it difficult to compare their AUCs with our study [7]. They reached an AUC of 0.87 with 15 parameters, which was the same as APACHE III, based on data from 23,446 ICU patients at Kentucky University Hospital in the USA during 1998–2007. It is clear that our AUC of 0.89 using data from only the first hour of admission is better than other models relying on more information using data recorded during the first 24 h. It is also worth mentioning that some other studies report AUCs on the training data and not the test data, something which should be discouraged due to the potential of achieving misleading AUCs by overfitting and therefore not being discussed here.

The main limitation of our study, as with all neural network models is that they can be viewed as “black box” models, i.e. there is little insight in how individual parameters contribute to the prediction. This problem is somewhat alleviated by ranking the predictors after their contribution to the total AUC. It is, however, inherent to many non-linear problems that the complex interactions found within the data are not easily expressed and interpreted. We believe that the primary aim of a good predictor is to just that: a good predictor (of mortality).

ICU prognostication is an ongoing process and will most likely improve significantly over the next decade due to an increasing amount of patient-level data. Based on this study, we believe logistic regression-based predictive modelling should be abandoned and instead replaced with machine learning algorithms like ANN.

## Conclusion

Our ANN model outperformed the SAPS 3 model (using the same data) in early (within 1 h of admission) prediction of 30-day mortality for intensive care patients in both AUC and calibration on a massive (217,289 admissions) dataset from the Swedish Intensive Care Registry. The superiority of our ANN model was also seen in the fact that an eight-parameter ANN model still outperformed the SAPS 3 model that uses over 40 parameters. The perhaps most important result was the fact that the ANN model was superior in correcting for the most important prognostic parameter, age. We thus encourage intensive care registries to use ANN models for short-term mortality predictions in quality control and research.

## Data Availability

The data is available from the Swedish Intensive Care Registry after anapproval process.

## Abbreviations

ANN: Artificial neural networks
APACHE: Acute Physiology And Chronic Health Evaluation
AUC: Area under the receiver operating characteristic curve
CNS: Central nervous system
EMR: Estimated mortality risk
FiO_2_: Fraction of inspired oxygen
GCS: Glasgow Coma Scale
ICU: Intensive care unit
OMR: Observed mortality rate
RLS85: Reaction Level Scale
ROC: Receiver operating characteristic curve
SAPS 3: The 3rd version of the Simplified Acute Physiology Score SIR: Swedish Intensive Care Registry

## References

[1] Metnitz PG, Moreno RP, Almeida E, Jordan B, Bauer P, Campos RA, Iapichino G, Edbrooke D, Capuzzo M, Le Gall JR (2005). Saps 3–from evaluation of the patient to evaluation of the intensive care unit. part 1: objectives, methods and cohort description. Intensive Care Med.

[2] Moreno RP, Metnitz PG, Almeida E, Jordan B, Bauer P, Campos RA, Iapichino G, Edbrooke D, Capuzzo M, Le Gall JR (2005). Saps 3–from evaluation of the patient to evaluation of the intensive care unit. part 2: development of a prognostic model for hospital mortality at ICU admission. Intensive Care Med.

[3] Haykin S (2009). Neural Networks and Learning Machines.

[4] Dawes T, de MA, Shi W, Fletcher T, Watson G, Wharton J, Rhodes C, Howard L, Gibbs J, Rueckert D, Cook S, Wilkins M, O’Regan D (2017). Machine learning of three-dimensional right ventricular motion enables outcome prediction in pulmonary hypertension: a cardiac mr imaging study. Radiology.

[5] Esteva A, Kuprel B, Novoa RA, Ko J, Swetter SM, Blau HM, Thrun S (2017). Dermatologist-level classification of skin cancer with deep neural networks. Nature.

[6] Pirracchio R, Petersen ML, Carone M, Rigon MR, Chevret S, van der Laan MJ (2015). Mortality prediction in intensive care units with the super ICU learner algorithm (SICULA): a population-based study. Lancet Respir Med.

[7] Kim S, Kim W, Woong Park R (2011). A comparison of intensive care unit mortality prediction models through the use of data mining techniques. Healthc Inform Res.

[8] Vincent J-L, Moreno R (2010). Clinical review: scoring systems in the critically ill. Crit Care (Lond Engl).

[9] Riskjusteringsmodeller Inom Svensk Intensivvård. https://www.icuregswe.org/globalassets/riktlinjer/riskjustering.pdf. Accessed 19 May 2019.

[10] Walther SM, Jonasson U, Gill H (2003). Comparison of the glasgow coma scale and the reaction level scale for assessment of cerebral responsiveness in the critically ill. Intensive Care Med.

[11] Abadi M, Agarwal A, Barham P, Brevdo E, Chen Z, Citro C, Corrado GS, Davis A, Dean J, Devin M, Ghemawat S, Goodfellow I, Harp A, Irving G, Isard M, Jia Y, Jozefowicz R, Kaiser L, Kudlur M, Levenberg J, Mané D, Monga R, Moore S, Murray D, Olah C, Schuster M, Shlens J, Steiner B, Sutskever I, Talwar K, Tucker P, Vanhoucke V, Vasudevan V, Viégas F, Vinyals O, Warden P, Wattenberg M, Wicke M, Yu Y, Zheng X. TensorFlow: Large-scale machine learning on heterogeneous systems. Software available from tensorflow.org. 2015. https://www.tensorflow.org/. Accessed 19 May 2019.

[12] Ioffe S, Szegedy C. Batch normalization: accelerating deep network training by reducing internal covariate shift. 2015. arXiv:1502.03167.

[13] Kingma DP, Ba J. Adam: A method for stochastic optimization. 2014. arXiv:1412.6980.

[14] Goodfellow I, Bengio Y, Courville A (2016). Deep learning, adaptive computation and machine learning.

[15] Srivastava N, Hinton G, Krizhevsky A, Sutskever I, Salakhutdinov R (2014). Dropout: a simple way to prevent neural networks from overfitting. J Mach Learn Res.

[16] Cheng-Yuan L, Wei-Chen C, Jiun-Wei L, Daw-Ran L (2014). Autoencoder for words. Neurocomputing.

[17] Fawcett T (2006). An introduction to roc analysis. Pattern Recognit Lett.

[18] DeLong ER, DeLong DM, Clarke-Pearson DL (1988). Comparing the areas under two or more correlated receiver operating characteristic curves: a nonparametric approach. Biometrics.

[19] Fenlon C, O’Grady L, Doherty ML, Dunnion J (2018). A discussion of calibration techniques for evaluating binary and categorical predictive models. Prev Vet Med.

[20] Lunneborg CE (2000). Data Analysis by Resampling: Concepts and Applications.

[21] R Core Team (2013). R: A Language and Environment for Statistical Computing.

[22] Cleveland WS, Grosse EE, Shyu WM, Chambers JM, Hastie TJ (1992). Local regression models. Statistical Models in S.

[23] Granholm A, Perner A, Krag M, Hjortrup PB, Haase N, Holst LB, Marker S, Collet MO, Jensen AKG, Møller MH (2018). Development and internal validation of the simplified mortality score for the intensive care unit (sms-icu). Acta Anaesthesiol Scand.

